# Supplementary material for: Environmental Burden of Childhood Disease in Europe
Source: Int J Environ Res Public Health. 2019 Mar 26;16(6):1084. doi: 10.3390/ijerph16061084 (PMC6466397; doi:10.3390/ijerph16061084)
Supplement: Supplementary file 1 [file ijerph-16-01084-s001.pdf]

Supplemental Material

Environmental Burden of Childhood Disease in Europe

David Rojas-Rueda, Martine Vrijheid, Oliver Robinson, Aasvang Gunn Marit, Regina Gražulevičienė, Remy Slama and Mark Nieuwenhuijsen

Index

A. Exposure data..2

Table 1. PM2.5 levels (g/m^3) in children les than 18 years old, by country in the EU28. 2

Figure 1. PM2.5 levels (g/m^3) in children les than 18 years old, by country in the EU28. 3

Table 2. Ozone levels in parts-per-billion (ppb), by country in the EU28. 4

Figure 2. Ozone levels in parts-per-billion (ppb), by country in the EU28........................5

Table 3. Secondhand smoke exposrue (%), by country in the EU28........................6

Figure 3. Secondhand smoke exposure (%), by country in the EU28. 7

Table 4. Micrograms of lead per deciliter of blood (μg/dL), by country in the EU28. 8

Figure 4. Micrograms of lead per deciliter of blood (μg/dL), by country in the EU28. 9

Table 5. Proportion of the total population living in homes with self-reported problems of damp, by country in the EU28. 10

Figure 5. Proportion of the total population living in homes with self-reported problems of damp, by country in the EU28. 11

B. Sensitivity analysis. 12

Table 6. Main results presented by heath outcome........................122

Table 7. Sensitivity analysis for health outcomes related with PM10. 122

Table 8. Sensitivity analysis for health outcomes related with Secondhand smoke. 13

Table 9. Sensitivity analysis for asthma related with dampness........................13

Table 10. Sensitivity analysis for asthma related with formaldehyde........................13

A. Exposure data.

Table 1. PM2.5 levels (g/m^3) in children les than 18 years old, by country in the EU28.

Country	Average	UCI	LCI
Austria	17,72	33,29	6,77
Belgium	19,08	34,66	7,81
Bulgaria	29,41	46,18	16,63
Croatia	23,16	39,29	11,09
Cyprus	21,13	37,22	9,4
Czech Republic	22,58	38,91	10,7
Denmark	11,73	26,29	3,18
Estonia	4,73	14,55	0,53
Finland	5,27	15,53	0,55
France	13,83	28,99	4,37
Germany	15,9	31,71	5,65
Greece	13,08	28,27	3,88
Hungary	28,35	45,16	15,22
Ireland	10,09	24,09	2,3
Italy	18,27	33,65	7,45
Latvia	17,22	33,05	6,67
Lithuania	19,77	35,38	8,36
Luxembourg	19,14	35,39	7,88
Malta	14,24	30,07	4,25
Netherlands	18,1	33,72	7,17
Poland	29,32	46,09	16,54
Portugal	10,55	24,39	2,48
Romania	22,48	38,8	10,55
Slovakia	23,88	40,44	11,67
Slovenia	21,07	37,44	9,44
Spain	11,22	25,48	2,91
Sweden	3,39	12,38	0,19
United Kingdom	13,87	29,11	4,48

PM2.5: particulate matter less than 2.5 micrometers of diameter.

Source: GBD 2016. GBD Results Tool User Guide. http://ghdx.healthdata.org/gbd-results-tool

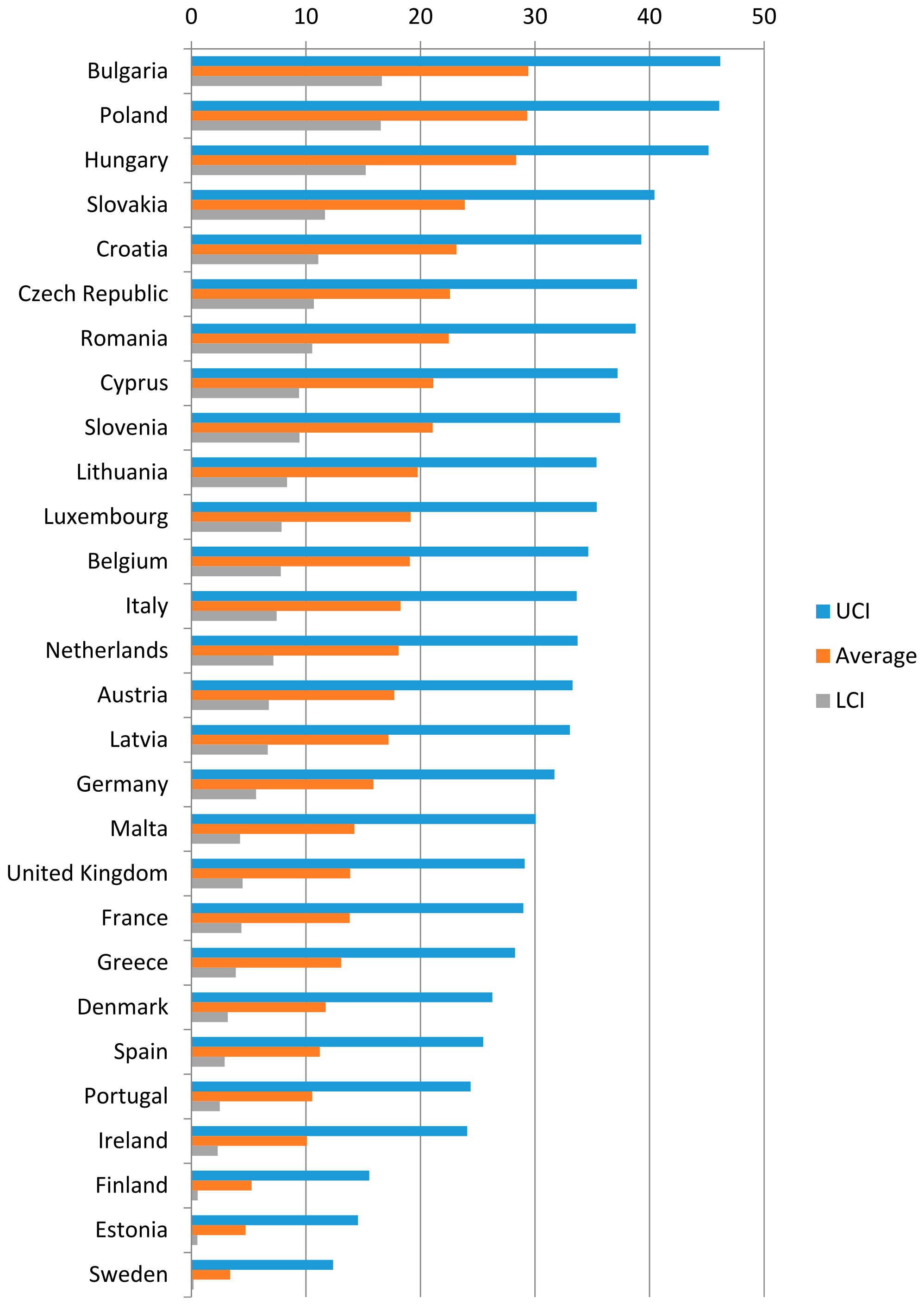

Figure 1. PM2.5 levels (g/m³) in children less than 18 years old, by country in the EU28.

PM2.5: particulate matter less than 2.5 micrometers of diameter. UCI: Upper confidence interval; LCI: Lower confident interval.
Source: GBD 2016. GBD Results Tool User Guide. http://ghdx.healthdata.org/gbd-results-tool

Table 2. Ozone levels in parts-per-billion (ppb), by country in the EU28.

Country	Average	UCI	LCI
Austria	40,76	74,03	14,52
Belgium	31,01	56,87	10,74
Bulgaria	51,84	92,27	18,53
Croatia	51,97	92,65	18,69
Cyprus	57,96	100	20,43
Czech Republic	38,4	69,77	13,89
Denmark	23,6	45,01	7,71
Estonia	18,97	37,36	5,66
Finland	16,77	33,6	5
France	35,96	66,01	12,77
Germany	33,28	61,04	11,64
Greece	61,13	100	22,13
Hungary	45,17	81,2	16,14
Ireland	19,13	37,73	5,74
Italy	69,88	100	25,58
Latvia	21,29	40,76	6,7
Lithuania	24,54	46,05	8,15
Luxembourg	31,9	59,59	10,89
Malta	59,78	100	21,55
Netherlands	29,25	54,34	9,85
Poland	33,82	62,08	11,82
Portugal	30,52	56,36	10,52
Romania	41,53	75,2	14,95
Slovakia	42,47	76,84	15,33
Slovenia	50,38	88,64	18,03
Spain	44,6	80,31	16,08
Sweden	15,76	31,82	4,7
United Kingdom	23,29	43,77	7,7

UCI: Upper confidence interval; LCI: Lower confident interval.

Source: GBD 2016. GBD Results Tool User Guide. http://ghdx.healthdata.org/gbd-results-tool

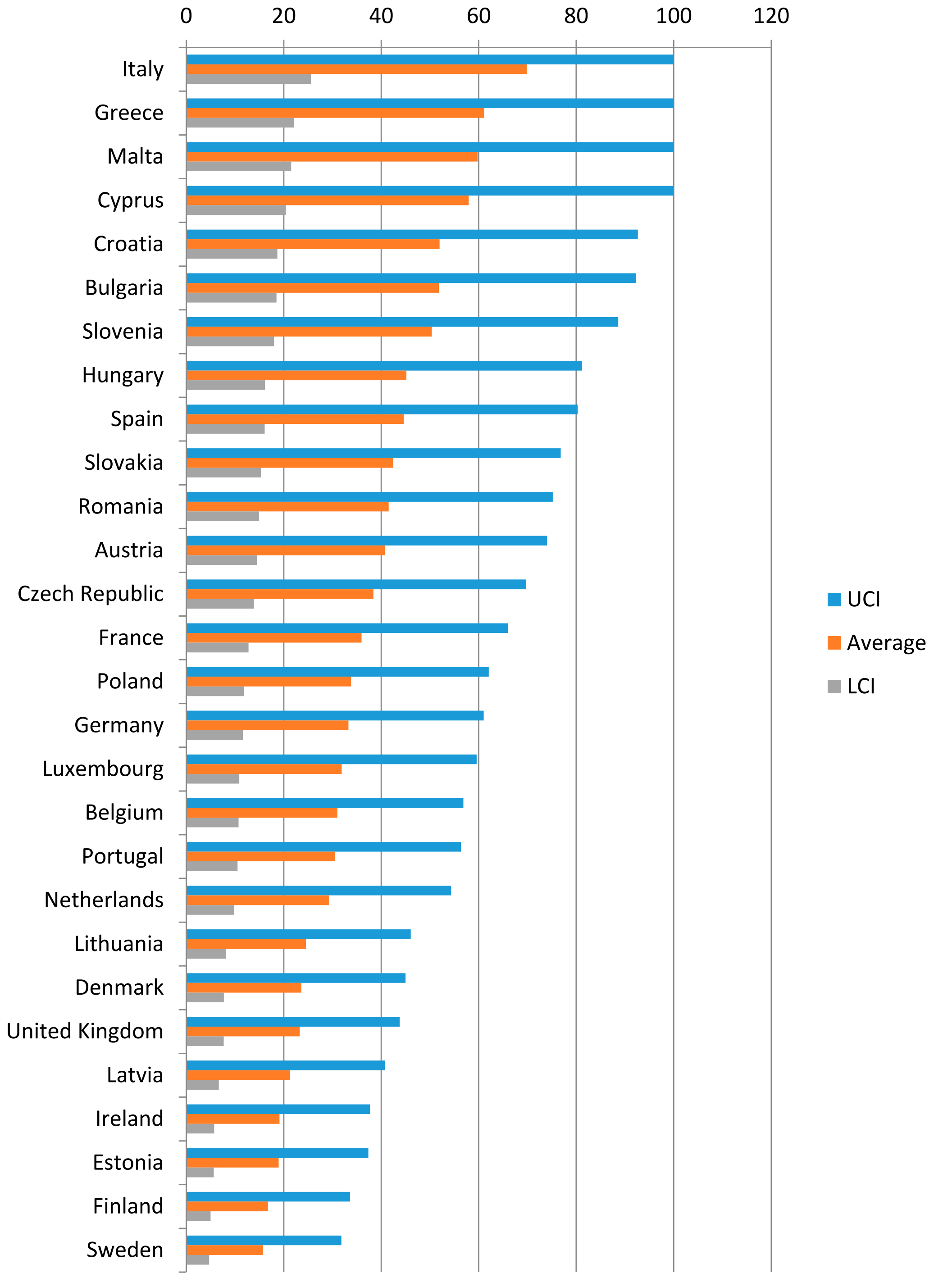

Figure 2. Ozone levels in parts-per-billion (ppb), by country in the EU28.

UCI: Upper confidence interval; LCI: Lower confident interval.
Source: GBD 2016. GBD Results Tool User Guide. http://ghdx.healthdata.org/gbd-results-tool

Table 3. Secondhand smoke exposrue (%), by country in the EU28.

Country	Average	UCI	LCI
Austria	49,8	52,41	47,28
Belgium	54,51	65,73	46,17
Bulgaria	74,87	80,4	68,4
Croatia	61,83	66,6	57,58
Cyprus	68,28	76	61,79
Czech Republic	59,02	64,31	54,45
Denmark	43,66	48,11	39,65
Estonia	57,94	65,18	51,7
Finland	34,8	38,14	31,34
France	46,93	50,01	43,93
Germany	47,99	53,03	43,56
Greece	69,63	74,71	64,89
Hungary	57,04	60,97	52,83
Ireland	45,58	48,27	43,08
Italy	46,25	49,75	42,74
Latvia	57,58	61,16	53,83
Lithuania	48,72	53,07	44,66
Luxembourg	60,96	72,98	51,84
Malta	60,81	74,1	50,35
Netherlands	48,43	54,89	43,19
Poland	56,23	60,11	52,48
Portugal	48,97	52,28	45,92
Romania	57,58	61,64	53,14
Slovakia	64,03	74,26	55,85
Slovenia	58,87	68,63	51,13
Spain	64,59	73,16	57,79
Sweden	33	36,02	30,13
United Kingdom	38,53	40,04	36,1

UCI: Upper confidence interval; LCI: Lower confident interval.

Source: GBD 2016. GBD Results Tool User Guide. http://ghdx.healthdata.org/gbd-results-tool

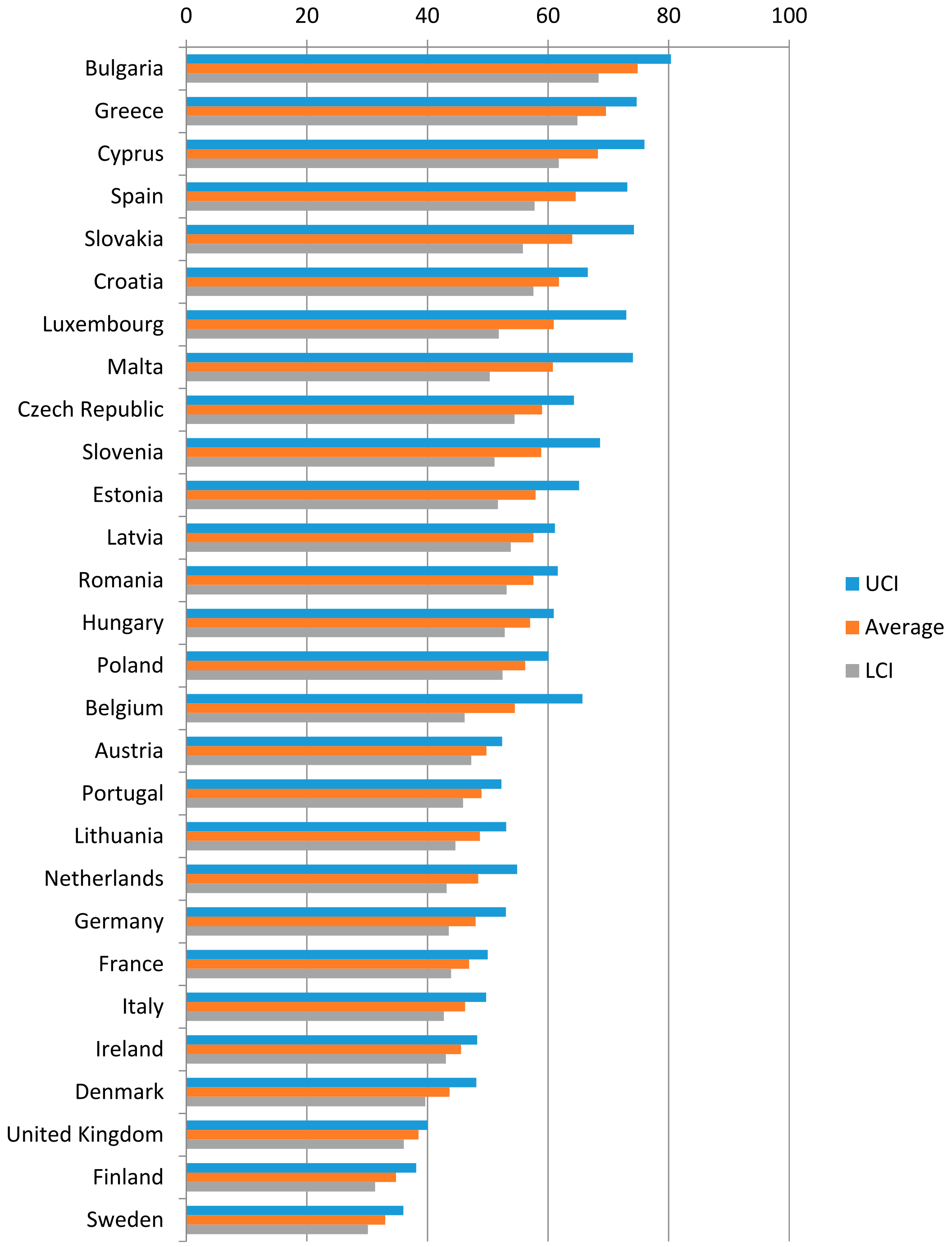

Figure 3. Secondhand smoke exposure (%), by country in the EU28.

UCI: Upper confidence interval; LCI: Lower confident interval.
Source: GBD 2016. GBD Results Tool User Guide. http://ghdx.healthdata.org/gbd-results-tool

Table 4. Micrograms of lead per deciliter of blood (µg/dL), by country in the EU28.

Country	Average	UCI	LCI
Austria	1,82	7,88	0
Belgium	12,35	24,8	3,92
Bulgaria	1,31	6,79	0
Croatia	0,97	5,67	0
Cyprus	5,19	14,24	0,5
Czech Republic	0,66	4,22	0
Denmark	2,83	10,32	0
Estonia	0,9	5,57	0
Finland	0,41	2,91	0
France	4,27	12,5	0,3
Germany	0,87	4,83	0
Greece	6,47	15,74	1,26
Hungary	1,98	9,05	0
Ireland	5,27	13,92	0,72
Italy	6,34	15,5	1,05
Latvia	1,15	6,33	0
Lithuania	1,01	5,73	0
Luxembourg	2,54	9,1	0,02
Malta	14,73	26,26	6,67
Netherlands	3,07	10,05	0,09
Poland	3,14	10,57	0,11
Portugal	7,23	17,21	1,55
Romania	2,18	9,02	0
Slovakia	0,95	5,41	0
Slovenia	0,84	5,05	0
Spain	8,77	19,25	2,3
Sweden	1,12	5,49	0
United Kingdom	1,49	6,1	0,06

UCI: Upper confidence interval; LCI: Lower confident interval.

Source: GBD 2016. GBD Results Tool User Guide. http://ghdx.healthdata.org/gbd-results-tool

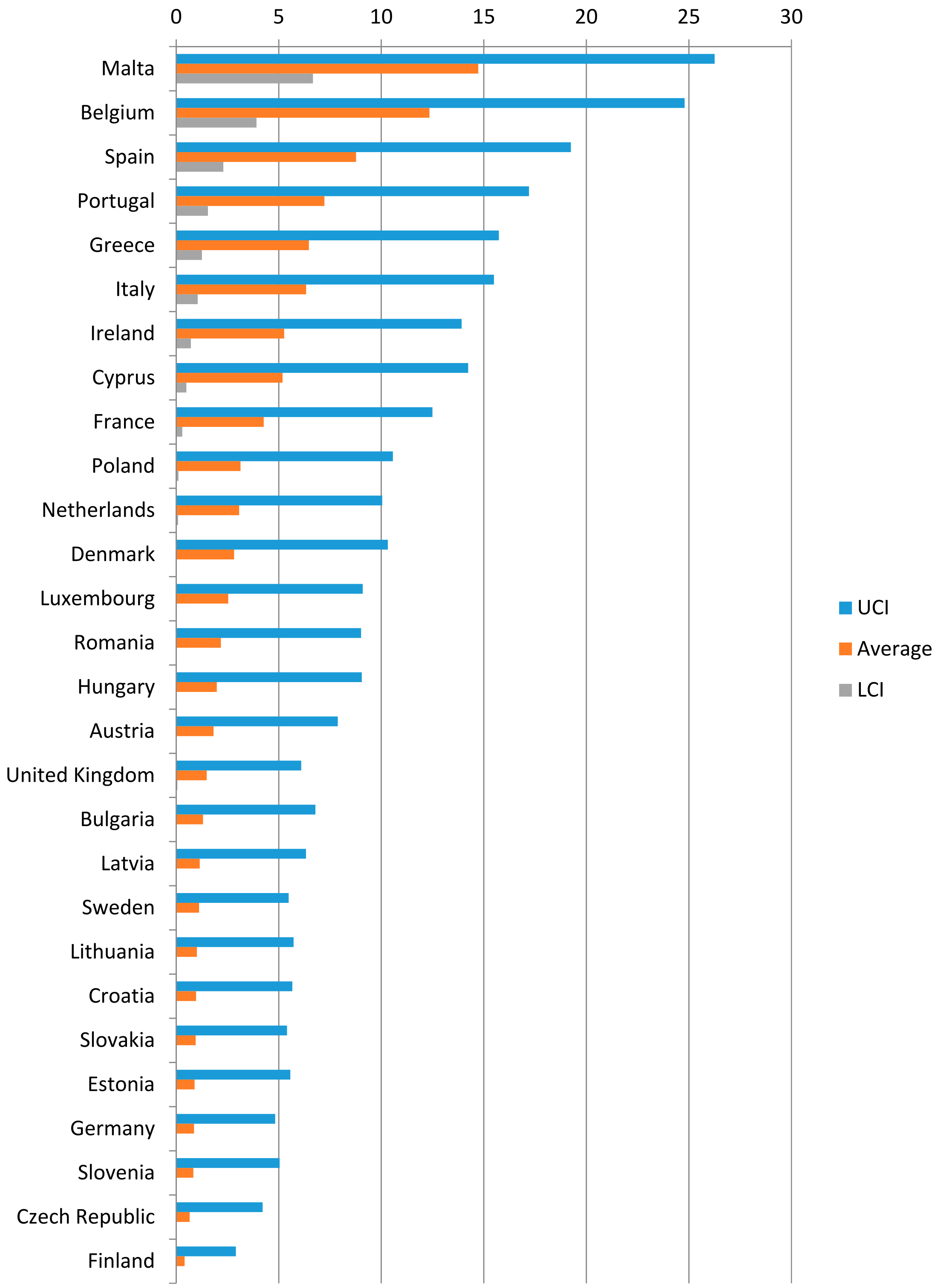

Figure 4. Micrograms of lead per deciliter of blood (µg/dL), by country in the EU28.

UCI: Upper confidence interval; LCI: Lower confident interval.
Source: GBD 2016. GBD Results Tool User Guide. http://ghdx.healthdata.org/gbd-results-tool

Table 5. Proportion of the total population living in homes with self-reported problems of damp, by country in the EU28.

Country	%
Austria	16,8
Belgium	21,1
Bulgaria	18,3
Croatia	11,1
Cyprus	28,5
Czech Republic	11,6
Denmark	21,5
Estonia	20,1
Finland	5,5
France	15,3
Germany	16,4
Greece	11,7
Hungary	30,6
Ireland	14,4
Italy	22,2
Latvia	29,9
Lithuania	16,9
Luxembourg	20,7
Malta	5,4
Netherlands	18,4
Poland	11,4
Portugal	21,7
Romania	18,5
Slovakia	10,5
Slovenia	30,9
Spain	17,8
Sweden	9,6
United Kingdom	21,9

Source: WHO Europe. Environment and Health Information System (ENHIS). 2016. https://gateway.euro.who.int/en/indicators/enhis_31-proportion-of-the-total-population-living-in-homes-with-self-reported-problems-of-damp/

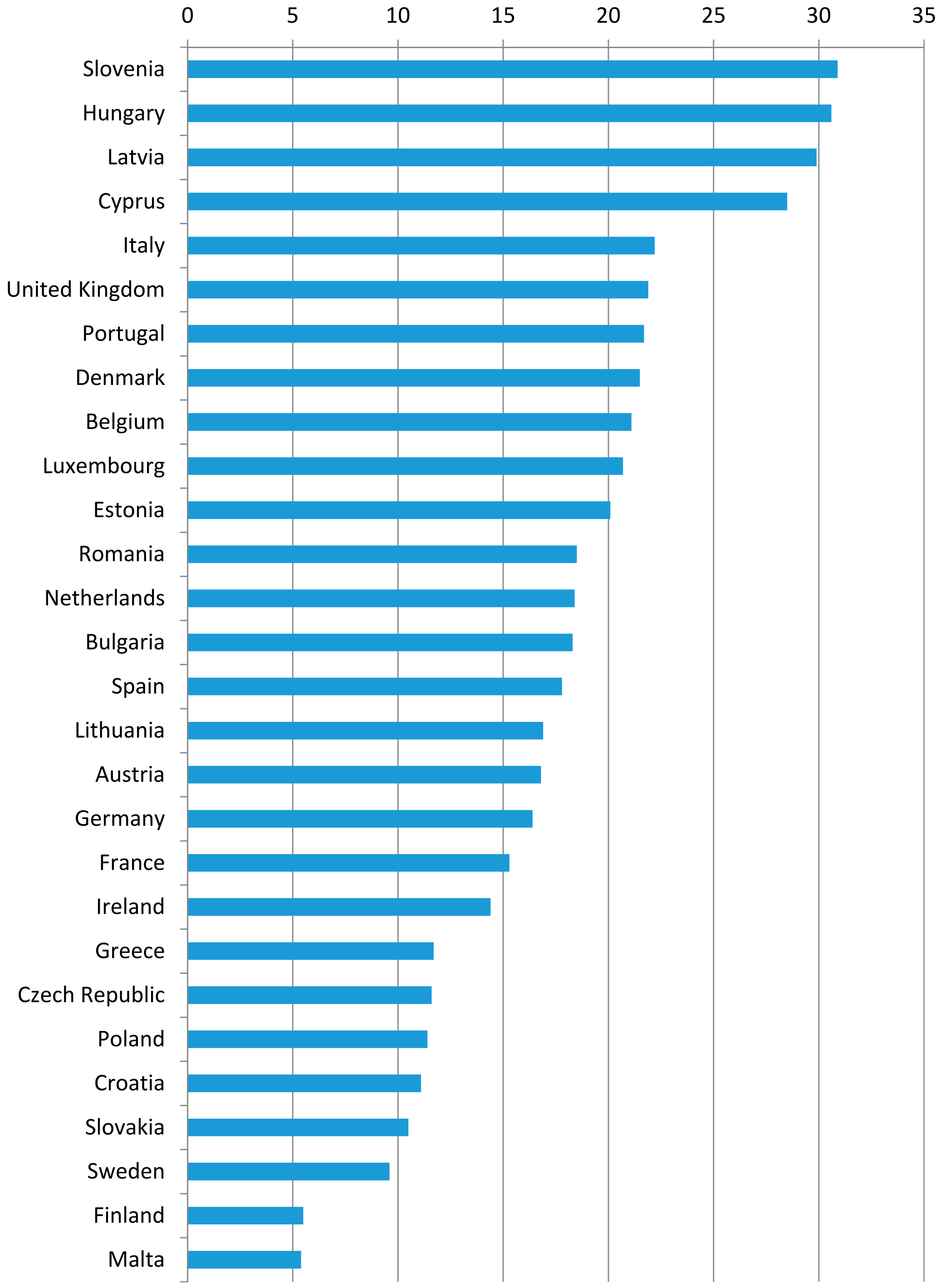

Figure 5. Proportion of the total population living in homes with self-reported problems of damp, by country in the EU28.

Source: WHO Europe. Environment and Health Information System (ENHIS). 2016. https://gateway.euro.who.int/en/indicators/enhis_31-proportion-of-the-total-population-living-in-homes-with-self-reported-problems-of-damp/

B. Sensitivity analysis.

Table 6. Main results presented by heath outcome.

Health outcome	Risk factor	Population	Cases	DALYs	LCI	UCI	DALYs/100.000 population	Total DALYs by outcome
**Mild mental retardation**	Lead	< 5 years	138 646	6 216	2 699	11 414	15.09	**6 216**
**Asthma**	PM10	5-18 years	43 402	13 904	462	15 181	17.80	
	Secondhand smoke	< 14 years	106 085	20 880	15 645	25 065	28.03	
	Dampness	< 14 years	65 815	12 954	3 022	31 646	17.39	
	Formaldehyde	< 3 years	423	33	4	83	0.60	**47 772**
**Low respiratory infections**	PM2.5	6-12 years	134 032	17 453	8 042	29 659	21.14	
	Secondhand smoke	< 5 years	142 530	9 728	5 942	14 040	37.79	**27 180**
**Infant mortality**	PM10	< 1 year	1 078	93 147	45 106	166 668	1 6324	**1 078**
**Otitis media**	Secondhand smoke	< 5 years	821 499	2 062	1 132	3 396	8.01	**2 062**
**Cough days**	Ozone	5-14 years	52 436 762*	10 057	902	10 177	19.78	**10 057**
**Low respiratory symptoms days**	Ozone	5-14 years	52 059 353*	14 122	760	14 207	27.78	**14 122**
**Total**								**210 777**

*Days with cough or other low respiratory symptoms; PM10: Particulate matter less than 10 micrometers of diameter; PM25: Particulate matter less than 25 micrometers of diameter; DALY: disability-adjusted life years; LCI: lower confidence interval; UCI: upper confidence interval.

Table 7. Sensitivity analysis for health outcomes related with PM10.

Sensitivity analysis	Total EU 28 DALYs	LCI	UCI
Asthma, main analysis	13 904	462	15 181
Asthma, assuming counterfactual of 1,9 mcg/m3*	18 681	420	3 272
Asthma, assuming counterfactual of 20 mcg/m3**	3 885	128	4 276
Using new exposure-response function (1.04 [1.00-1.08], per 2 mcg/m3)***	45 098	0	46 582
Infant mortality, main analysis	93 147	45 106	166 668
Infant mortality, assuming counterfactual of 1,9 mcg/m3*	124 794	68 921	195 378
Infant mortality, assuming counterfactual of 20 mcg/m3**	30 499	16 195	49 725

*As suggested by Turner M, et al. for the ACS study;

**As suggested by the Air quality guidelines of the WHO 2006.

*** As suggested by Khreis H, et al 2017.

Table 8. Sensitivity analysis for health outcomes related with Secondhand smoke.

Sensitivity analysis	Total EU 28 DALYs	LCI	UCI
Asthma, main analysis	20 880	15 645	25 065
Asthma, using the minimum percentage of SHS reported in EU 28*	12 848	9 124	16 301
Low respiratory infections, main analysis	9 728	5 942	14 040
Low respiratory infections, using the minimum percentage of SHS reported in EU 28*	6 867	6 127	7 341
Otitis media, main analysis	2 062	1 132	3 396
Otitis media, using the minimum percentage of SHS reported in EU 28*	1 168	1 015	1 338

* The Secondhand smoke minimum exposure reported (15%) in the EU 28 (Finland).

Table 9. Sensitivity analysis for asthma related with dampness.

Sensitivity analysis	Total EU 28 DALYs	LCI	UCI
Dampness, main analysis	12 954	3 022	31 646
Using mould as a exposure (instead of dampness)	11 470	1 023	33 667

Table 10. Sensitivity analysis for asthma related with formaldehyde.

Sensitivity analysis	Total EU 28 DALYs	LCI	UCI
Main analysis	33	4	83
Using 60 mcg/m3 as a threshold	1 667	187	4 510
